# Are reviewers suggested by authors as good as those chosen by editors? Results of a rater-blinded, retrospective study

**DOI:** 10.1186/1741-7015-4-13

**Published:** 2006-05-30

**Authors:** Elizabeth Wager, Emma C Parkin, Pritpal S Tamber

**Affiliations:** 1 Sideview, Princes Risborough, HP27 9DE, UK; 2 BioMed Central, London, W1T 4LB, UK

## Abstract

**Background:**

*BioMed Central *(*BMC*) requires authors to suggest four reviewers when making a submission. Editors searching for reviewers use these suggestions as a source. The review process of the medical journals in the *BMC series *is open – authors and reviewers know each other's identity – although reviewers can make confidential comments to the editor. Reviews are published alongside accepted articles so readers may see the reviewers' names and recommendations.

Our objective was to compare the performance of author-nominated reviewers (ANR) with that of editor-chosen reviewers (ECR) in terms of review quality and recommendations about submissions in an online-only medical journal.

**Methods:**

Pairs of reviews from 100 consecutive submissions to medical journals in the *BMC series *(with one author-nominated and one editor-chosen reviewer and a final decision) were assessed by two raters, blinded to reviewer type, using a validated review quality instrument (RQI) which rates 7 items on 5-point Likert scales. The raters discussed their ratings after the first 20 pairs (keeping reviewer type masked) and resolved major discrepancies in scoring and interpretation to improve inter-rater reliability. Reviewers' recommendations were also compared.

**Results:**

Reviewer source had no impact on review quality (mean RQI score (± SD) 2.24 ± 0.55 for ANR, 2.34 ± 0.54 for ECR) or tone (mean scores on additional question 2.72 ANR vs 2.82 ECR) (maximum score = 5 in both cases). However author-nominated reviewers were significantly more likely to recommend acceptance (47 vs 35) and less likely to recommend rejection (10 vs 23) than editor-chosen reviewers after initial review (p < 0.001). However, by the final review stage (i.e. after authors had responded to reviewer comments) ANR and ECR recommendations were similar (65 vs 66 accept, 10 vs 14 reject, p = 0.47). The number of reviewers unable to decide about acceptance was similar in both groups at both review stages.

**Conclusion:**

Author-nominated reviewers produced reviews of similar quality to editor-chosen reviewers but were more likely to recommend acceptance during the initial stages of peer review.

## Background

Identifying peer reviewers is an important part of an editor's job. This task is especially difficult for general journals that cover a wide range of subject areas, many of which will be outside the editor's own area of expertise. If reviewers are unsuitable (e.g. do not know enough about the subject or are biased) this might affect the outcome of the peer-review process (i.e. decisions about acceptance). The choice of reviewer may also affect the quality of reviews and how opinions are expressed (i.e. the tone of the review and whether it is courteous).

Some journals ask authors to suggest potential reviewers but little is known about the effects of such a policy. Concerns have been raised that reviewers nominated by authors will not be as critical as those chosen by editors. (As an editorial in the *BMJ *put it 'the worry about using nominated reviewers is that peer review will become a cosy process of endorsement by friends and colleagues'.)[1] At the time of the present study, only one other study had been published, but it used a non-validated scoring system and was done at a journal which used anonymous reviewing and did not routinely ask authors to suggest reviewers.[2] We therefore decided to compare the quality of reviews received from reviewers suggested by authors with those from reviewers chosen by editors in a journal that routinely uses author-nominated reviewers and operates an open peer-review system (i.e. authors and reviewers know each other's identities).

### Background: the *BioMed Central *review process

When submitting an article, authors are asked to suggest four possible reviewers. All submissions are done online and the reviewer suggestion fields are compulsory.

Authors are advised that reviewers 'should be experts in their field of study, who will be able to provide an objective assessment of the manuscript'. They are also asked to exclude anyone who has published with any of the authors within the last five years and anyone who works at the authors' research institution(s).

Editors searching for reviewers use authors' suggestions as one source for identifying potential reviewers. Editors aim not to use more than one author-nominated reviewer (ANR) without one editor-chosen reviewer (ECR). Two reviews are usually obtained for each submission. Reviewers' names are shown on the reviews, although there is also a facility for reviewers to make confidential comments to the editor. Reviews are published alongside accepted articles so readers also know who the reviewers were and what recommendations they made.

Reviews are submitted using an online form. Reviewers are asked for their comments on the submission and must choose between the options: accept without revisions; accept with discretionary revisions; accept after minor essential revisions; unable to decide; reject because too small an advance; or reject because not scientifically sound.

## Methods

Pairs of reviews from 100 submissions to medical journals in the *BMC series *were assessed by two raters (EW and PST). We included the 100 most recent submissions which had a final decision about publication and for which the journal had received reviews from one ANR and one ECR (this comprised submissions from October 2003 to March 2004). Raters were blinded to reviewer type (ANR or ECR) but not to reviewer identity. Reviews were assessed using the Review Quality Instrument (RQI).[3] This rates seven features of reviews on 5-point Likert scales (see Table 2 for content). We also included an eighth item on review tone which was used in an earlier version of the RQI but later omitted; this item rates reviews on a 5-point scale from abusive to courteous. The overall score for each review was the mean of the seven item scores. Results from the tone question were analysed separately and did not contribute to the overall score. The two raters discussed scores after the first 20 pairs (keeping reviewer type masked) and resolved major discrepancies in scoring and interpretation to improve inter-rater reliability.

The primary objective was to compare the quality of reviews received from ANRs with those from ECRs as shown by the mean RQI (7-item total) score. A difference in review quality of at least 10% (0.4/4) was defined, *a priori*, as editorially significant, in line with previous studies. Secondary objectives were to compare recommendations about acceptance/rejection, review tone and timeliness.

### Statistical methods

To detect a difference of 10% (α = 0.05, β = 0.10, SD = 1.2) we required 94 manuscripts to analyse. As distributions of scores and differences were close to a normal distribution we used paired t-tests to compare evaluations of ANR and ECR review quality. Reviewers' recommendations on publication were compared using the chi-squared test.

## Results

There was no statistically significant difference in review quality for ANRs and ECRs as measured by the mean RQI score (Table 1). There was also no significant difference in review tone. However, ANRs were more likely to recommend acceptance and less likely to recommend rejection than ECRs after initial review (Table 1). Recommendations about acceptance were similar at the final review stage (i.e. after authors had responded to reviewers' comments) for ANRs and ECRs, although slightly more ANRs than ECRs stated that they were unable to decide on acceptance or rejection. There was no significant difference between the groups in the time taken to supply a review.

Mean scores for the individual RQI items are shown in Table 2. The raters observed consistent patterns in the scores for different items and therefore did a *post hoc *analysis of this. The lowest item scores were associated with discussing the originality of the research, providing evidence to substantiate comments, and commenting on authors' interpretation of their results. Reviewers tended to perform better on providing constructive comments, identifying methodological strengths and weaknesses, and assessing the writing and organization of submissions. The difference in scores between the three items with the highest mean scores (constructive comments, methodology review, and assessing writing and organization) and the three with the lowest mean scores (research originality, providing evidence, and commenting on authors' interpretation) was statistically significant (p = 0.04).

## Discussion

Our findings suggest that ANRs produce reviews of similar quality to ECRs. However, ANRs were significantly more likely to recommend acceptance and less likely to recommend rejection than ECRs during the initial stages of peer review. The significance of this observation depends on how editors regard reviewer recommendations. In journals that rely on reviewer judgements to a great extent (e.g. always accepting submissions if two, or a majority of, reviewers recommend this) use of ANRs could affect a submission's chance of acceptance. However, in many journals, although editors base their decision on the reviewers' comments, they do not necessarily follow the reviewers' recommendations about acceptance or rejection. Indeed, it has been pointed out that it is not a good idea to 'count votes' since 'one would need to have at least six reviewers, all favouring publication or rejection for their votes to yield a statistically significant conclusion'.[5] If ANRs tend to recommend publication more often than ECRs, journals that use ANRs should try to ensure that the proportion of ANRs and ECRs is the same for all papers, so that submissions are treated equally.

ANRs' unwillingness to reject papers and their tendency to state that they were unable to decide (despite, in some cases, producing a critical review) may be a feature of using ANRs within an open peer-review system. A reviewer known personally to the author may feel more constrained about rejecting a submission, despite having produced an objective and critical review. Requiring reviewers to sign their reviews may increase this phenomenon. One study comparing open and anonymous review found that anonymous reviewers rejected 8% more manuscripts than identified reviewers, however this difference was not statistically significant.[4]

While it may seem reasonable to assume that ANRs are more likely to know authors personally than ECRs this may not necessarily be true. Authors may select reviewers by their reputation or publication record and editors may unknowingly select reviewers with personal links to the authors. In our study, reviewers were not told who had selected them, so ANRs were probably unaware of their status unless authors had informed them. Although authors are asked to suggest reviewers without obvious close links (such as recent joint publications or working at the same institution), they do not always follow these instructions and editors rely on reviewers to inform them if they have a conflict of interest. One aspect that our study did not address is how distinct ANRs and ECRs really are. It would be interesting to follow up with a study in which editors selected reviewers before viewing the authors' recommendations and measuring how often the editors identified the same potential reviewers as the authors.

Our study was done in a series of journals that use online, open peer-review. We cannot tell to what extent our findings are generalizable to journals that use different peer-review systems such as anonymous review. Our findings across a range of biomedical specialties may also have masked variations between research fields (for example there may be differences between large and small disciplines where the chances of authors knowing both the ANR and the ECR may vary).

When we started our research, only one other study on ANRs had been published.

Earnshaw *et al *compared the reviews from ANRs with those from ECRs in a surgery journal that used anonymous reviewing. However, in this case, the authors were told that reports from ANRs would not be used to assess their submission. A non-validated 5-item scoring scheme was used, with each item scored 1–4. Earnshaw *et al *concluded that ECRs produced more critical reviews than ANRs. However the actual difference between the groups was small, and the difference only reached statistical significance for assessments of scientific importance (mean scores: ANR 2.34 vs ECR 2.56, p = 0.009) and decision (2.51 vs 2.75, p = 0.029). These differences, despite reaching statistical significance, do not reach the threshold suggested by Van Rooyen *et al *that an editorially meaningful difference should be at least 10% (in this case 0.3/3).

Our findings of no important difference in review quality between ANRs and ECRs is also supported by a study undertaken at around the same time as ours by Schroter *et al *at the *BMJ*.[6] The *BMJ *study assessed 329 submissions to 10 biomedical journals and found mean RQI scores of 2.58 for ANR and 2.64 for ECR (our figures were 2.24 and 2.34 respectively). Reviewers could choose between recommending acceptance, resubmission or rejection. Schroter *et al *found that ANRs were more likely to recommend acceptance (57% vs 46%) and less likely to recommend rejection (13% vs 24%) than ECRs. This is a similar pattern to our findings, although the proportion of reviewers recommending rejection is higher, probably reflecting the actual rejection rates and editorial policies at the *BMJ *journals.

The time taken to supply reviews was also virtually identical in our study and that from the *BMJ*. Schroter *et al *report a median of 18 days for both groups, while we observed medians of 18 and 17 days for ANRs and ECRs.

We observed that mean total review quality scores and mean scores for individual questions were generally low (<3 (= midpoint) out of a maximum of 5 in each case). However, the range is similar to that observed by Schroter *et al *who also found average scores below the midpoint.[6]

Although the RQI was not designed to compare different components of reviews, and this was a *post hoc *analysis, the scoring ranks assigned by the two independent raters were consistent, suggesting that this analysis was valid. We noted that reviewers performed best on aspects that help authors improve the quality of their submission (e.g. providing constructive comments) while they tended to perform less well on aspects that help editors select papers (e.g. commenting on the originality of the research). This may be because most reviewers have more experience as authors than as editors. Our observations are similar to those of van Rooyen *et al *who compared anonymous with identified reviewers using the RQI. They also reported the highest scores for constructive comments and the lowest score for commenting on the originality of the research.[3] These observations might be useful when designing guidance or training for reviewers.

## Conclusion

Author-nominated reviewers (ANRs) produced reviews of similar quality to editor-chosen reviewers (ECRs). However, ANRs were significantly more likely to recommend acceptance at initial review, and slightly more likely to state that they were unable to decide between acceptance and rejection on final review, than ECRs. We conclude that the use of ANRs is unlikely to materially affect the quality of reviews received, however it could affect acceptance decisions if journals rely heavily on reviewer recommendations.

## Authors' contributions

EW had the original idea for the study, which was designed jointly by EW, PST and ECP. EW and PST did the ratings, ECP identified reviews for inclusion and collected and analysed the data. EW prepared the first draft of the paper, which was critically revised by PST and ECP.

## Competing interests

This study was done with the cooperation of BioMed Central but without formal funding. At the time of the study PST and ECP were employees of BioMed Central and received a fixed salary.

## Pre-publication history

The pre-publication history for this paper can be accessed here:



## Figures and Tables

**Table 1:** Review quality (mean total RQI), review tone, reviewers' recommendations on acceptance, and timeliness of supplying review for reviewers nominated by reviewers (ANRs) or chosen by editors (ECRs)

	**Author-nominated reviewers (ANR)**	**Editor-chosen reviewers (ECR)**	***p*-value**
*N*	100	100	
Mean RQI (± SD)	2.24 ± 0.55	2.34 ± 0.54	0.18
Tone	2.72 ± 0.48	2.82 ± 0.52	0.94

Recommendation: first review (N=%)			
accept	47	35	<0.001
reject	10	23	
can't decide	43	42	

Recommendation: final review (after revision)			
accept	65	66	0.47
reject	10	14	
can't decide	25	20	

Median time to supply review (days) (range)	18 (1–48)	17 (1–64)	

**Table 2:** Mean scores for individual items of the Review Quality Instrument (RQI) for all reviewers (ANRs and ECRs)

**RQI item**		Mean score*	**SD**
1	Assessing importance of research question	2.34	0.78
2	Assessing originality	1.87	0.89
3	Identifying strengths and weaknesses of methods	2.41	0.73
4	Providing comments about writing and presentation	2.39	0.87
5	Providing constructive comments	2.73	0.81
6	Providing evidence to support comments	2.18	0.86
7	Commenting on authors' interpretation of results	2.28	0.75

*Mean of both raters
